# A case of endoscopic combined oval forceps removal of a rectal smooth curved-surface foreign body

**DOI:** 10.1055/a-2665-7148

**Published:** 2025-08-20

**Authors:** Zongjing Hu, Huihui Zhou, Yaowen Zhang

**Affiliations:** 1 562122 Department of Gastroenterology, Affiliated Hospital of Jining Medical University, Jining, China; 2 562122 Endoscopy Department, Affiliated Hospital of Jining Medical University, Jining, China

A 35-year-old male patient was admitted due to a self-inserted rectal foreign body for 1 day. The foreign body is silicone material. Abdominal CT showed a rectal foreign body and no evidence of perforation ( Fig. 1 **a–c** ). After admission, endoscopic foreign body removal was performed. Inserting the gastroscope transanally into the rectum, a red smooth curved-surface foreign body was visualized ( Fig. 2 **a** ), measuring approximately 5.7 cm × 4.7 cm ( Video 1 ). Repeated application of snare, rat-tooth forceps, and lithotripter inner cores to remove the foreign body failed due to the smooth, curved surface of the foreign body. So, we applied endoscopic combined oval forceps ( Fig. 3 ) to clamp and fix the foreign body, which was large and slippery, and slipped out during transanal retraction; a small amount of silicone-like debris was clamped down. The foreign body slips off and bounces back to the rectosigmoid junction. Rubber gloves were placed above the foreign body to prevent it from advancing to the sigmoid colon. The gastroscope crosses over the foreign body and pulls it to the lower rectum after flipping the endoscope. Finally, we adjusted the angle of the oval forceps, firmly clamped the foreign body, and successfully extracted it through slow rotation and traction. The rectal mucosa was slightly damaged without bleeding or perforation ( Fig. 4 ). The patient recovered well and was discharged 2 days after the operation. Follow-up 1 week after the operation, no significant discomfort was observed.

**Fig. 1:**
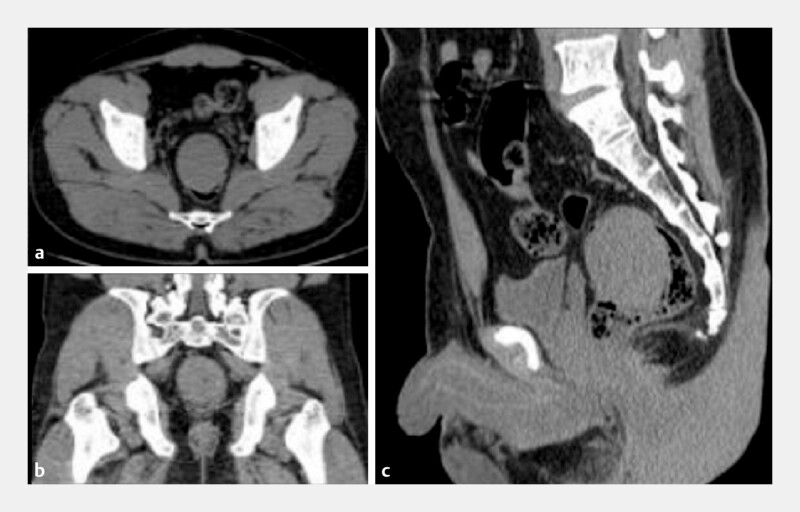
**a–c** Abdominal CT showed a rectal foreign body and no evidence of perforation.

**Fig. 2:**
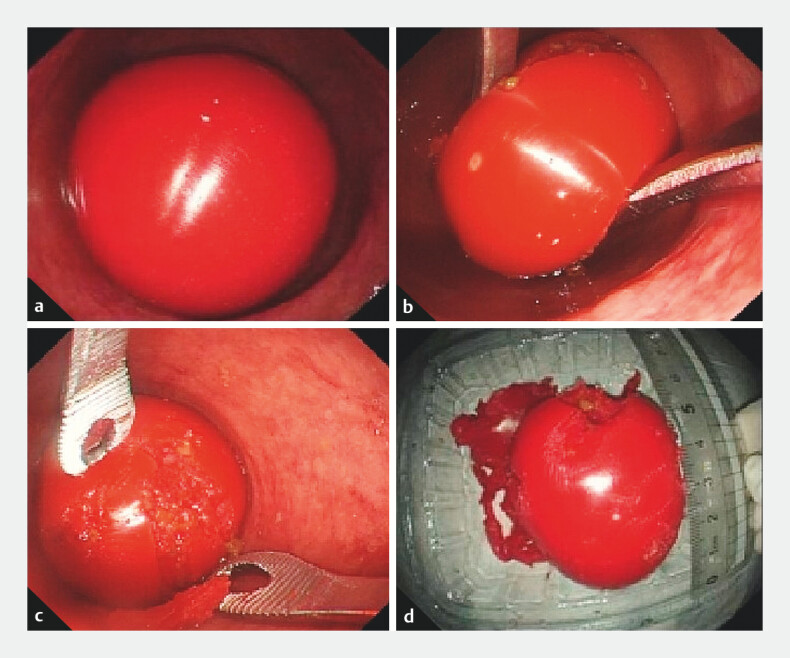
**a** Endoscopy revealed a red smooth curved-surface foreign body in the rectum, measuring approximately 5.7 cm × 4.7 cm. **b, c** Under direct endoscopic visualization, application of oval forceps to clamp the foreign body. **d** The foreign body was successfully removed.

**Fig. 3:**
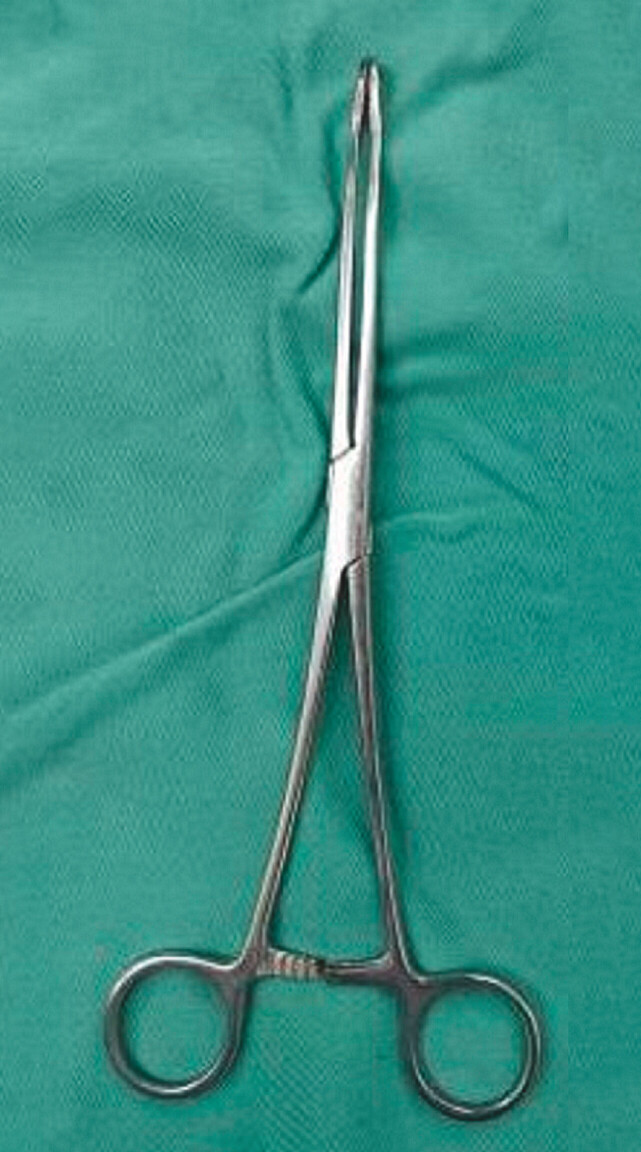
Oval forceps.

**Fig. 4:**
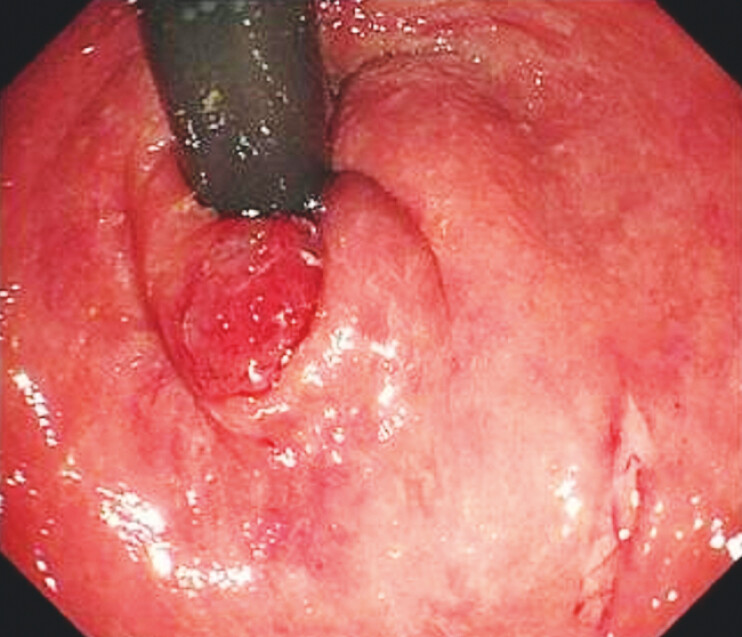
The rectal mucosa was slightly damaged.

A case of endoscopic combined oval forceps removal of a rectal smooth curved-surface foreign body.Video 1

There are multiple techniques for extracting rectal foreign bodies 1 2 3 , and less invasive initial approaches are recommended. The jaws of the oval forceps are highly conformable to smooth curved-surface foreign bodies 4 . Combined with the visualization of the endoscope, it is a minimally invasive and effective method for removing smooth curved-surface foreign bodies. To prevent the upward movement of foreign bodies, rubber gloves can be useful. Endoscopic combined oval forceps removal of rectal foreign bodies reduces the risk of complications such as bleeding, perforation, and infection, with faster patient recovery and avoidance of open surgery.

Endoscopy_UCTN_Code_TTT_1AQ_2AH

## Notes

Endoscopy E-Videos https://eref.thieme.de/e-videos
*E-Videos* is an open access online section of the journal *Endoscopy* , reporting on interesting cases and new techniques in gastroenterological endoscopy. All papers include a high-quality video and are published with a Creative Commons CC-BY license. Endoscopy E-Videos qualify for HINARI discounts and waivers and eligibility is automatically checked during the submission process. We grant 100% waivers to articles whose corresponding authors are based in Group A countries and 50% waivers to those who are based in Group B countries as classified by Research4Life (see: https://www.research4life.org/access/eligibility/ ). This section has its own submission website at https://mc.manuscriptcentral.com/e-videos .
